# Empirical Kinetic Models for the CO_2_ Gasification of Biomass Chars. Part 1. Gasification of Wood Chars and Forest Residue Chars

**DOI:** 10.1021/acsomega.1c04577

**Published:** 2021-10-10

**Authors:** Gábor Várhegyi, Liang Wang, Øyvind Skreiberg

**Affiliations:** † Institute of Materials and Environmental Chemistry, Research Centre for Natural Sciences, P.O. Box 286, Budapest 1519, Hungary; ‡ SINTEF Energy Research, Postboks 4761 Torgarden, NO-7465 Trondheim, Norway

## Abstract

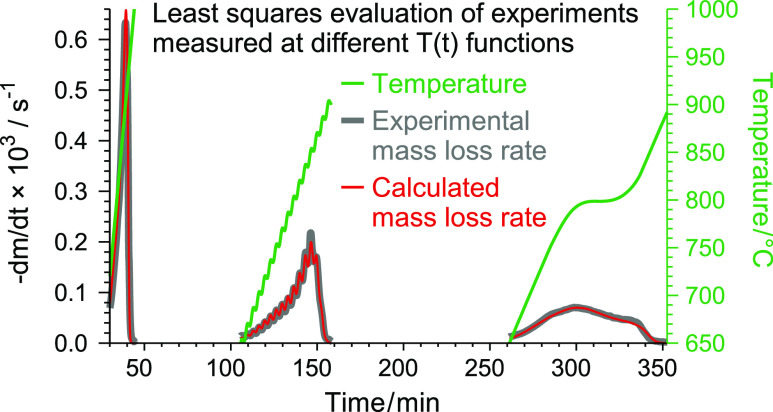

The gasification kinetics of charcoals and biomass chars is complicated by several factors, including chemical and physical inhomogeneities, the presence of mineral matter, and the irregular geometry of the pore structure. Even the theoretically deduced gasification models can only provide empirical or semiempirical descriptions. In this study, an empirical kinetic model from the earlier works of the authors was adapted for the CO_2_ gasification of biomass chars. It is based on a versatile polynomial approximation that helps to describe the dependence of the reaction rate on the progress of the conversion. The applicability of the model was tested by the reevaluation of 24 thermogravimetric analysis (TGA) experiments from earlier publications. The adjustable parameters of the model were determined by the method of least squares by evaluating groups of experiments together. Two evaluation strategies were tested. In the regular evaluations, the same kinetic parameters were employed for all the experiments with a given sample. The use of experiments with modulated and constant reaction rate (CRR) temperature programs made it possible to employ another approach too, when the preexponential factor was allowed to vary from experiment to experiment. The latter approach allows a formal kinetic description of the differences in the thermal deactivation of the samples caused by different thermal histories as well as of some inevitable systematic errors of the TGA experiments. The evaluations were carried out by both approaches, and the results were compared. The evaluations were based on 12 experiments. As a test, each evaluation of the study was repeated with only 8 experiments. The results of the latter test calculations indicated that the information content of the employed experiments is sufficient for the evaluation approaches of this work.

## 1 Introduction

The char + CO_2_ reaction is an important partial reaction in nearly all biomass gasification processes.^1^ It is considered to be the slowest of the main reactions in gasification; its rate is much lower than that of the char + H_2_O reaction under identical experimental conditions; hence, the char + CO_2_ partial reaction is frequently the rate-determining step of gasification.^2^ The CO_2_ gasification of chars may be a viable way for utilization of various biomass wastes and residues.^3−6^ Besides, chars with a favorable pore structure and/or large surface area can also be produced by CO_2_ gasification.^7^ The kinetics of the char + CO_2_ reaction is important in the understanding and modeling of the listed processes. It is a fast-growing field; a recent review reported 510 publications from 2014 until 2020 of which a major part dealt with the kinetics of this reaction too.^8^ Earlier detailed reviews are also available.^9,10^

When the system is far from equilibrium and the CO concentration is not high, the kinetics of the char + CO_2_ reaction is generally described by equations like
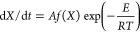
1where *X* is the conversion which varies from zero to one as the reaction proceeds. This quantity is usually denoted by α in the literature of thermal analysis,^11^ and the α notation was employed in the earlier papers of the present authors too. *A* and *E* are the preexponential factor and the activation energy, respectively. For high-purity idealistic carbons, the *f*(*X*) function can be derived theoretically. Several theoretical models have been developed since the publication of the seminal works of Bhatia, Perlmutter, and Gavalas.^12,13^ The gasification of a real char, however, differs from the ideal behavior by various complicating factors, including chemical and physical inhomogeneities, the presence of the mineral matter, the catalytic effect of some of the inorganic elements, and the irregular geometry. Accordingly, the theoretical *f*(*X*) functions in the literature serve only as semiempirical or empirical models for the description of most real chars. The preexponential factor obviously depends on the partial pressure of CO_2_. This dependence is usually approximated by a power function.^1−3,5,8,9^ For atmospheric pressure experiments, it is more convenient to write the corresponding equation with the volume concentration of CO_2_, *C*_CO_2__, as
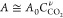
2where ν is a reaction order.^5^ (If partial pressure were used in eq 2, then the dimension of *A*_0_ would depend on the actual value of ν: it would be Pa^–ν^ s^–1^.)

Thermogravimetric analysis, TGA, is a useful method to study the kinetics of the corresponding processes in the kinetic regime due to its high precision.^9^ It can be carried out with isothermal and nonisothermal temperature programs. The isothermal experiments are usually carried out so that the sample is heated to the desired temperature in an inert gas and then the gas is switched to CO_2_ or to a CO_2_–inert gas mixture. However, the stabilization of the CO_2_ concentration in the furnace is not instantaneous. Naredi et al.^14^ showed that a complete flushing of the inert gas from their TGA apparatus typically took around 20 min. They studied the CO_2_ gasification of coal chars at 850 °C. Similar results were achieved later for the CO_2_ gasification of graphite by Zhang et al.^15^ The increase of the CO_2_ concentration after the gas switch results in an increasing reaction rate and may produce a false maximum in the apparent *f*(*X*) function. It is possible that a considerable part of the existing gasification literature is based on such artifacts. A more reliable way is to switch the gas below the gasification temperature and include the heat-up section too in the kinetic evaluation. We do not need the mathematical simplicity of the isothermal evaluations at the high processing level of computers and computing methods in our age. Any kinetic equation of type 1 at any *T*(*t*) function can easily be solved numerically, and the model parameters can be found by the method of least squares. Besides, there is no need to stick to the simple isothermal and linear *T*(*t*) functions because a higher variation of the temperature programs increases the available information in the experiments.^5,6,16,17^

Várhegyi proposed versatile empirical models that can be used as *f*(*X*) functions in eq 1 and tested them on 85 earlier thermogravimetric experiments from studies on the thermal decomposition (pyrolysis) of 16 samples.^18^ The model can be employed with constant *E* values as well as with empirical *E*(*X*) functions. The first approach is simpler and also gave appropriate results. In a subsequent work, this type of modeling was extended for the description of the combustion of biomasses and chars, and it was tested on 38 TGA experiments.^19^ The present work examines the applicability of this type of modeling on the CO_2_ gasification of biomass chars. Here again, earlier experiments are reevaluated, and particular care is taken to check the reliability of the results. The model in the notation of the present work has the form
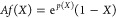
3where *p*(*X*) is a polynomial and its coefficients are the model parameters. Equation 3 follows directly from the combination of eqs 5 and 6 in the work of Várhegyi et al., 2020.^19^ The term (1 – *X*) in eq 3 ensures that *Af*(*X*) is zero at the end of the reaction for any polynomial coefficients. Substituting eq 3 into eq 1, we get
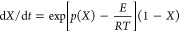
4

Equation 4 can be solved numerically at any *T*(*t*). The method of least squares can provide such polynomial coefficients for which the fit is the best between the experimental data and their counterparts calculated from the model.^18,19^ The obtained *Af*(*X*) function can be factorized into an *A* value and an *f*(*X*) function by the normalization of *f*(*X*). A plausible way is to assume that the maximum of *f*(*X*) is 1, as it was done by Várhegyi et al., 1996, in a char combustion work with another empirical model.^16^ Nevertheless, it is much simpler to normalize the *f*(*X*) functions so that their values would be 1 at a selected *X* value. In the present work, the selection of *X* = 0 resulted in particularly simple formulas for the calculation of *A*, as shown in Section 2.3.

In the present study, the fit quality, the shape of the obtained *f*(*X*) functions, and the reliability of the results were examined in detail in the ways outlined above. Besides, some aspects that may arise in any kinetic modeling of the thermoanalytical experiments were also examined.

## 2 Methods

### 2.1 Samples and Experiments

Such TGA experiments were reevaluated in the present work that had been evaluated earlier by other models:

(i) 12 TGA experiments carried out on a wood char and a forest residue char at two CO_2_ concentrations.^5^
(ii) 12 TGA experiments carried out on wood and forest residue chars that were prepared by a slow and a fast pyrolysis process.^6^

Linear, modulated, and constant reaction rate (CRR) experiments were carried out in these publications to increase the information content of the experiments. Note that a suitable kinetic model should describe well the gasification at any *T*(*t*) temperature programs. The particularities of the experiments are briefly summarized at the beginning of each section treating calculations on the given data set. More details can be found in the original publications.^5,6^

### 2.2 Evaluation by the Method of Least Squares and Characterization of the Fit Quality

Most works in this field evaluated the experimental counterparts of the conversion, *X*^obs^, in some way
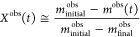
5where *m*^obs^(*t*) is the normalized sample mass at time *t,* while *m*_*i*nitial_^obs^ and *m*_final_^obs^ are its values at the start and at the end of the gasification, respectively. In real world experiments, however, it is frequently impossible to obtain reliable estimates for *m*_initial_^obs^ because the start of the gasification partially overlaps with other processes, such as devolatilization and the release of the chemisorbed species. Accordingly, our previous char gasification studies were based on the evaluation of the −d*m*/d*t* values and—for compatibility—this practice was followed in the present work too. Such values were searched for the unknown model parameters which minimized the difference between the experimental (−d*m*/d*t*)^obs^ and the predicted (−d*m*/d*t*)^calc^ data, that is, the objective function *of* was minimized
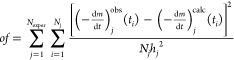
6where *N*_exper_ is the number of experiments evaluated together, *N*_*j*_ is the number of *t*_*i*_ time values in experiment *j*, and *h*_*j*_ is the highest experimental point on the given experimental curve. The division by *h*_*j*_^2^ is for normalization.

The experimental −d*m*/d*t* values were obtained by approximating the *m*^obs^(*t*) values by smoothing splines.^20^ The root-mean-square (rms) difference between the original *m*(*t*) and the smoothing spline was typically much below 1 μg. Such small differences do not introduce considerable systematic errors into the least-squares kinetic evaluations.^21^

The numerical solution of the kinetic equation, eq 4, provides d*X*^calc^/d*t* values which are proportional to the −d*m*^calc^/d*t* values
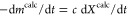
7

If *m*_initial_^obs^ is known with reasonable precision, then *c* can be calculated from eq 5 as
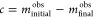
8

This was the case in the work of Wang et al., 2013.^5^ Otherwise, *c* is an adjustable parameter that can be determined together with the other parameters by the method of least squares.^6^

The obtained fit quality can be characterized separately for each of the experiments. For this purpose, the relative deviation (reldev, %) was used. The rms difference between the observed and calculated values is expressed as the percent of peak maximum
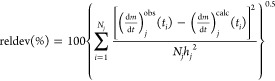
9

The fit quality for a given group of experiments is characterized by the rms of the corresponding reldevs. For example, the rms reldev for 12 experiments is denoted as reldev_12_.

### 2.3 The Polynomial in the Model and the Preexponential Factor

Based on our experience with this type of modeling,^18,19^ fifth-order polynomials were chosen for eqs 3 and 4. There is a strong compensation effect between the high-order coefficients of the polynomials, as explained by figures in the Supporting Information. It can be eliminated by two simple transformation steps. The first is to introduce an *x* variable which varies in the [−1,1] interval
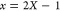
10

Introducing *x* into eq 3
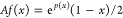
11where *p*(*x*) is the polynomial *p*(*X*) expressed by powers of *x* instead of *X*
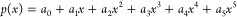
12

As mentioned in the Introduction, the experiments define *A* and *f*(*x*) together in the kinetic equation. If we wish to factorize the *Af*(*x*) term to separate *A* and *f*(*x*) factors, normalization of *f*(*x*) is needed. For example, we can normalize the starting point of *f*(*x*) to be 1, that is, we can assume that *f* = 1 at *x* = −1. Equations 11 and 12 then yield 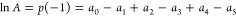
13

The numerical properties of the model can further be improved if *p*(*x*) is expressed by Chebyshev polynomials of the first kind.^22^ Only a few simple lines are needed for this purpose in the programs as shown in the Supporting Information. Equation 12 is then replaced by
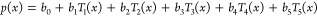
14

If this way is followed, then coefficients *b* are determined by the method of least squares instead of coefficients *a* of eq 12. Equation 13 has a similarly simple form in this case too^22^
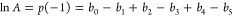
15

Note that there is no need to transform back the *p*(*x*) polynomials to the powers of *X*. At any *X* value, we can calculate the corresponding *x* by eq 10 and evaluate *p*(*x*) either by eq 14 or by eq 12. See further details in the Supporting Information.

### 2.4 Evaluation with Identical and Scattering Parameters

In regular evaluations, all the parameters are identical in the experiments belonging to the same sample at a given concentration of the reacting gas. However, the reactivity may have some variation from experiment to experiment, which can formally be described by a variation of the preexponential factor, as explained in Section 3.3. Equations 13 and 15 show that it can be achieved by allowing *a*_0_ or *b*_0_ to vary from sample to sample, while the rest of the polynomial coefficients are identical in the given set of experiments. The extent of this variation can be characterized by the deviation of the ln *A* values from their mean. One can calculate either the rms or the average of the absolute values of these deviations. The two approaches gave similar values in the present work. Hence, the simpler one, the mean absolute deviation, was used. The obtained deviation of ln *A* was converted to the common logarithm (via a division by ln 10) and was denoted as δlog_10_*A* in the tables.

### 2.5 Computing Methods

The least squares evaluations were carried out by simple but safe numerical methods. The experimental temperature values were connected by linear interpolation, and eq 1 was solved by a Runge–Kutta method for each experiment in each [*t*_*i*–1_, *t*_*i*_] interval.^22^ The minimization of the objective function was carried out by a variant of the Hooke–Jeeves method. The Hooke–Jeeves method is a slow but simple and dependable direct search algorithm.^23^ Further details can be found about the employed numerical methods in the earlier works of Várhegyi et al.^18,24^

Most evaluations in the present work included the finding of common *E* values for different samples. This was carried out by evaluations in a grid of predefined *E* values and selecting the optimal one. The process was described in an earlier publication in its Section 3.4 and Figure 7.^6^ This procedure resulted in simpler programs and better numerical stability than the other approaches tested. However, the results obtained in this way were based on thousands of least squares evaluations using eq 6. Therefore, many calculations obviously needed some automation by scripts and other means, as described earlier.^18^

## 3 Results

### 3.1 Gasification of Wood and Forest Residue Chars

**Figure 1:**
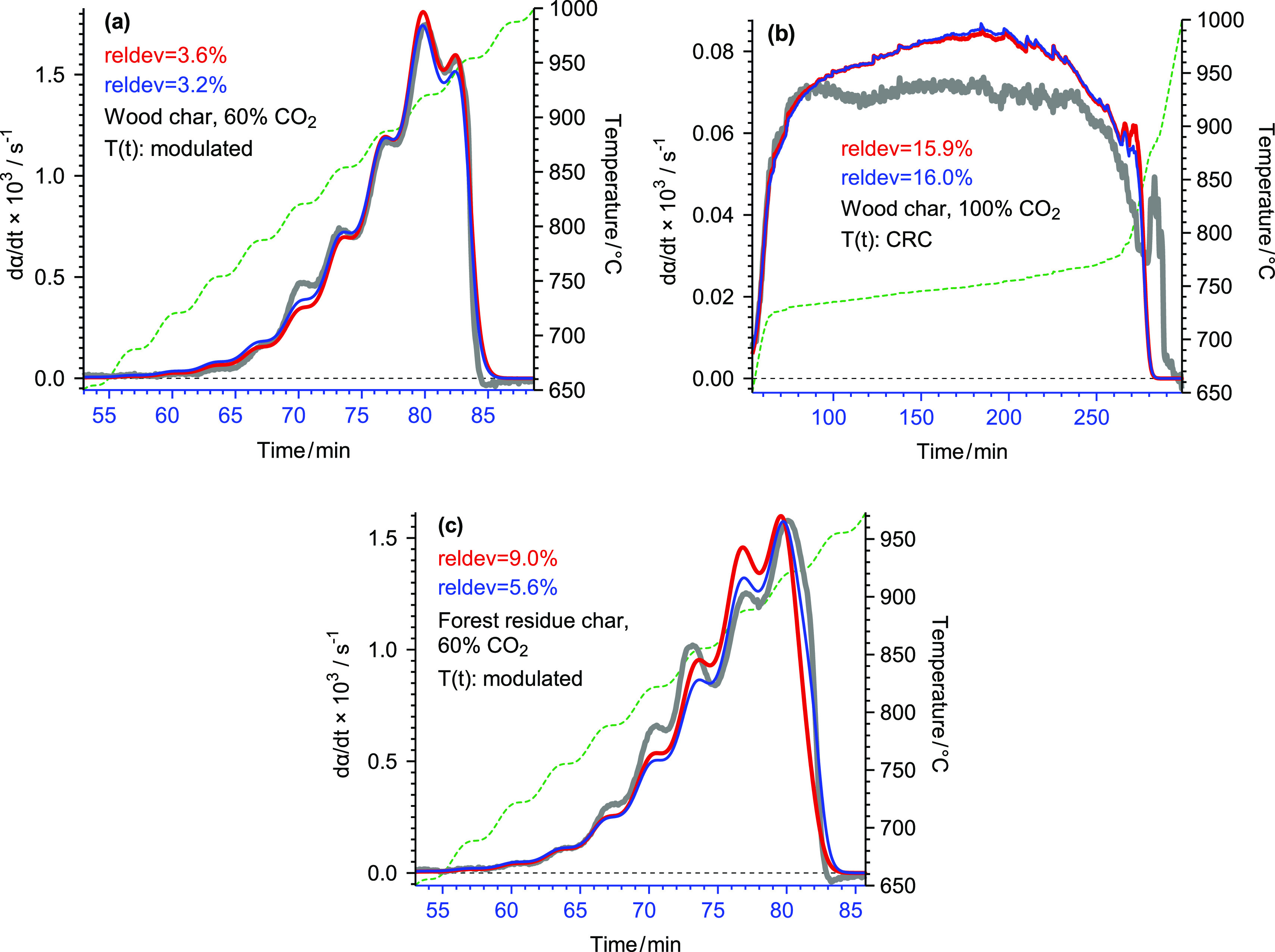
The best (a), the worst (b), and a typical fit quality (c) in Evaluation 1.2. The (−d*m*/d*t*)^calc^ curves of this evaluation are denoted by red color. The (−d*m*/d*t*)^calc^ data from Evaluation 1.4 are indicated by blue lines for comparison. The thick gray lines and the green dashed lines represent the experimental curves and the observed *T*(*t*) data, respectively.

The first part of this section contains results that were obtained via the reevaluation of the experiments of Wang et al., 2013,^5^ by the present model. A brief summary is given here about these experiments to facilitate the reading of the treatment. More information can be found in the original work of Wang et al.^5^ The chars were prepared from spruce wood and from a forest residue. Three temperature programs had been used: (i) linear *T*(*t*) with a heating rate of 10 °C/min; (ii) modulated *T*(*t*), where sinus waves with 5 °C amplitudes and 200 s wavelength were superposed onto a 10 °C/min linear *T*(*t*); and (iii) “CRR” *T*(*t*), when the TGA equipment regulated the heating of the samples so that the mass loss rate fluctuated around a preset limit of around 0.08 μg/s. The v/v concentration of CO_2_ was 0.6 and 1 in the gas flow. 12 experiments were available. The shape of the −d*m*/d*t* curves was irregular and the dependence of the shape of the curves on the CO_2_ concentration was atypical, as shown in Figure 1 from the work of Wang et al., 2013.^5^ The initial sample mass was low to avoid heat and mass transfer problems. The kinetic evaluation was based on *n*-order kinetics with respect to *X* and ν-order kinetics with respect to the volume concentration of CO_2_. In the present study, we used the experiments that were carried out with around 1 mg initial sample mass.

### 3.2 Results of the Reevaluations of the Experiments of Wang et al., 2013^5^

Table 1 lists the main characteristics of the evaluations, as explained below. For comparison, we took Evaluation 2 from the work of Wang et al.^5^ because it was the nearest to the possibilities of the present work. In that case, a common *E* value was searched for both samples, while the *f*(*X*) function was different for the wood and the forest residue chars. This evaluation is Evaluation 1.1 in Table 1. The model used in 2013 included eq 2, and the ν values were determined together with the other model parameters.

**Table 1:** Results Based on the Experimental Data of Wang et al., 2013^5^

no.	evaluation^a^	*N*_exper_^b^	reldev_*N*_, %^c^	*E*, kJ/mol	δlog_10_ *A*	aver. ν
1.1	**2013**	12	10.2	220	0	0.88
1.2	regular	12	8.5	220	0	0.68
1.3	regular	8	8.5	219	0	0.72
1.4	*A* varies	12	7.1	200	0.05	0.76
1.5	*A* varies	8	7.2	198	0.06	0.73

a The values in the first line characterize an evaluation of Wang et al., 2013,^5^ while the rest of the table belongs to the present study. The terms “regular” and “*A* varies” denote the two approaches explained in Section 2.4.

b 12 experiments were available for the evaluations. As a test, the evaluations were also carried out with only 8 experiments.

c reldev_*N*_ is reldev_12_ for Evaluations 1.1, 1.2, and 1.4, and it is reldev_8_ for evaluations 1.3 and 1.4. (See further details in the text.)

The second row in Table 1 is an evaluation by the methods of the present work. The main difference from Evaluation 1.1 is the use of a more versatile formula for the empirical approximation of *f*(*X*). The term “regular” in the second column is explained in the next paragraph. The fit quality is a bit better due to the higher versatility of the formulas describing *f*(*X*) in the present work. The red lines in Figure 1 illustrate the best, the worst, and a typical fit quality obtained by this evaluation. The ν values were calculated after evaluation from the obtained *A* values: the *A* values were different at the two CO_2_ concentrations and their ratio provided ν through eq 2. The average of the two ν values was considerably lower than the value obtained in 2013. This result is consistent with the other evaluations of the present work: the average ν values in Evaluations 1.2–1.5 scattered around a mean of 0.72 with a standard deviation of 0.03. The preexponential factors themselves are not listed in the tables because they strictly follow the values of the activation energies due to the well-known compensation effect between *E* and ln *A*. However, the Supporting Information contains the preexponential factors for the evaluations based on 12 experiments.

A question of key importance is as follows: can the given experimental data provide sufficient information for the evaluation by this model? To check it, all evaluations of the work were carried out with fewer experiments. In the present data set, the linear experiments were omitted for this purpose so that two experiments remained for each char at a given CO_2_ concentration: a modulated experiment and a particularly slow CRR experiment. Essentially, the same *E* and the same fit quality were obtained in this way, while the average ν showed some alteration. (Here, the obtained ν, 0.72, fell in the middle of the aforementioned 0.72 ± 0.03 range.)

### 3.3 Allowing the Variation of the Preexponential Factor within a Group of Experiments

The thermal deactivation (annealing) of chars has been known for decades.^25^ Accordingly, the reactivity of the chars depends on their thermal history prior to the gasification. This dependence can be approximated by a change in the preexponential factor.^25,26^ Some annealing differences may develop during the heat up of the samples within the TGA at different heating programs. Besides, the annealing continues during the gasification as well, and hence, the difference in the temperature programs may affect the reactivity during the gasification as well. The experimental errors of the TGA apparatus can also influence the kinetic results because the measured temperature usually differs from the actual temperature within the sample.^27^ The sampling from inhomogeneous chars may also cause some variation from experiment to experiment. It is possible to formally describe the factors outlined above by allowing variations in the preexponential factors, while the rest of the parameters should be kept identical for a given sample to avoid ill-conditioning.^6,16^ The modulated and CRR experiments employed in our work provided sufficient information to carry out such evaluations, as discussed in Section 4.

In the present case, the variation of *A* resulted in a 9% decrease in the activation energy, as Evaluation 1.4 shows in Table 1. The variation of the preexponential factor resulted in a lower reldev_12_ value than those of Evaluations 1.2 and 1.3, but it is still much higher than the values obtained on other samples as discussed in the next section. The fit quality of Evaluation 1.4 is indicated by blue lines in Figure 1. The difference between the red and blue lines was considerable only in Figure 1c.

The variation of the preexponential factors highly increases the number of unknown parameters to be determined by the method of least squares. Nevertheless, an evaluation carried out on fewer experiments resulted in nearly identical *E* and a similar fit quality. The δlog_10_*A* and average ν values were also nearly identical in Evaluations 1.4 and 1.5.

### 3.4 Comparison of the *f*(*X*) Functions Obtained by Different Approaches

**Figure 2:**
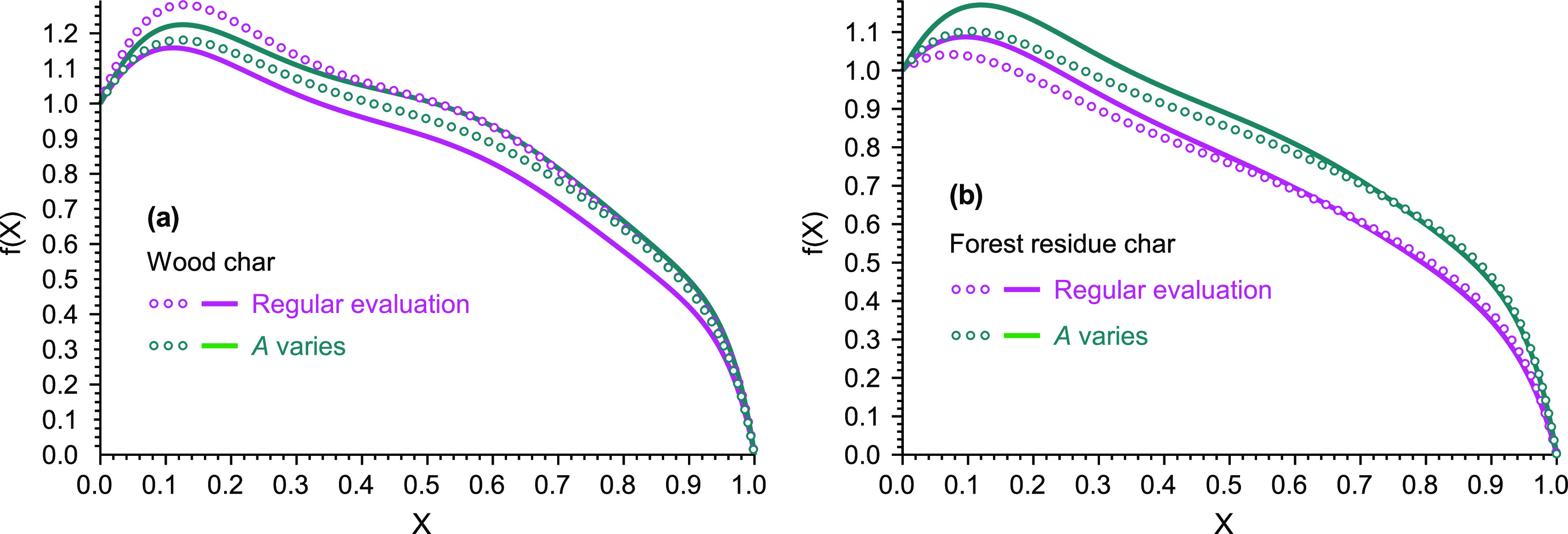
Comparison of the *f*(*X*) functions obtained for wood char (a) and forest residue char (b). Thin solid lines indicate the curves from the evaluation of all available experiments. Circles represent the curves obtained from fewer experiments, as described in the text.

The *f*(*X*) functions belonging to Evaluations 1.2–1.5 are shown in Figure 2. The values show roughly 10% alterations in each panel of Figure 2, while the main features of the obtained curves are similar: a notable peak maximum at the beginning of the curves followed by a shoulder-like part, which is more marked in Figure 2a.

### 3.5 Gasification of Chars from Slow and Fast Pyrolysis

In the rest of Section 3, results are presented that were obtained via the reevaluation the experiments of Wang et al., 2018.^6^ A brief summary is given here about these experiments. Wang et al. studied chars prepared from spruce pellets, S, and forest residue pellets, R.^6^ Part of the experiments was carried out so that the chars formed inside the TGA apparatus during the employed heating programs: 20 °C/min heating; a modulated *T*(*t*) when sinus waves were superposed to 5 °C/min heating; and CRR heating. Note that the heating rates in the linear and modulated *T*(*t*) programs differed from the ones employed in the earlier work of Wang et al., 2013.^6^ The gas flow was 100% CO_2_. Another part of the experiments was carried out with chars that were prepared in a drop-tube reactor heated till 1200 °C with a high heating rate. These chars were denoted by S1200 and R1200. The gasification of samples S and R occurred in a wide temperature range and the corresponding kinetic description assumed two parallel reactions that were intended to describe the more reactive and the less reactive parts of the samples.^6^ The kinetics of chars S1200 and R1200 were simpler. The preexponential factor was allowed to scatter for reasons outlined in Section 3.3.

An evaluation with a common *E* was selected for comparison with the results of the present work. This is Evaluation 2.1 in Table 2. Figure 7 in the paper of Wang et al., 2018, shows how the optimal *E* = 222 kJ/mol value was found.^6^

**Table 2:** Results Based on the Experimental Data of Wang et al., 2018^6^

no.	evaluation^a^	*N*_exper_^b^	reldev_*N*_^c^	*E*, kJ/mol	δlog_10_ *A*
2.1	**2018**	12	3.7	222	0.05
2.2	A varies	12	3.0	231	0.04
2.3	A varies	8	2.4	230	0.04
2.4	A varies	8	2.2	246	0.01
2.5	regular	12	4.2	266	0
2.6	regular	8	2.8	271	0
2.7	regular	8	2.7	267	0

a The values in the first line characterize an evaluation of Wang et al., 2018.^6^

b 12 experiments were available for the evaluations. As a test, the evaluations were also carried out with only 8 experiments.

c reldev_*N*_ is reldev_12_ for evaluations 1, 2, and 5, and it is reldev_8_ for evaluations 3, 4, 6, and 7. (See further details in the text.)

### 3.6 Evaluations of the Experiments from 2018 with Varying *A*

**Figure 3:**
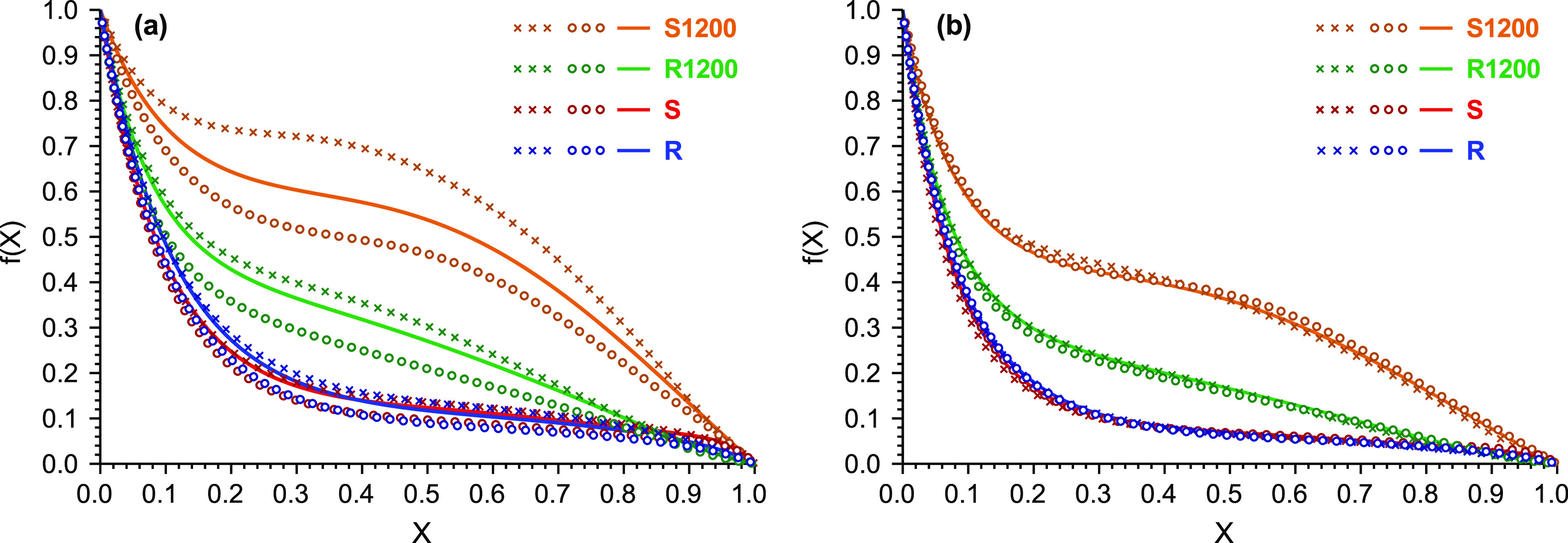
Comparison of the *f*(*X*) functions obtained at varying *A* (a) and by regular evaluations (b) from the experiments of Wang et al., 2018.^6^ The solid lines belong to the evaluation of all available experiments. Symbols × represent Evaluations 2.3 and 2.6. The circles correspond to Evaluations 2.4 and 2.7.

In the work of Wang et al., 2018,^6^ the *c* parameters were common for the experiments of a given sample, while *A* was allowed to vary from experiment to experiment. The reasons for a varying *A* are summarized above in Section 3.3. Evaluation 2.2 in Table 2 corresponds to this approach with the modeling of the present paper. Each of the four samples had its own *f*(*X*) function determined by eq 14, which is plotted in Figure 3. It is interesting to observe that samples S and R had nearly identical *f*(*X*). This does not mean, however, that samples S and R have similar reactivities: the peak temperatures of the corresponding −(d*m*/d*t*)^calc^ curves showed a difference of ca. 25 °C at a 20 °C/min heating rate. In this case, the reactivity difference is expressed mainly by the preexponential factors which can be found in the Supporting Information. (The forest residue chars have higher reactivities than the wood chars for reasons outlined in our earlier work.^5,6^)

The next two evaluations in Table 2 were carried out to test the information content of the experiments. In Evaluation 2.3, the CRR experiments were omitted; hence, the determination of the model parameters was based on the linear and modulated *T*(*t*) experiments. In this case, practically, the same activation energy was obtained as in Evaluation 2.2. In Evaluation 2.4, the 20 °C/min experiments were omitted, and the evaluation was based on the experiments with modulated and CRR *T*(*t*). Here, the obtained *E* differed by ca. 6% from the result of Evaluation 2.2. The *f*(*X*) functions obtained from Evaluations 2.3 and 4 were close to the results of Evaluation 2.2 for samples S and R, while appreciable differences appeared for samples S1200 and R1200. Nevertheless, the shapes of the obtained *f*(*X*) functions were similar in Evaluations 2.2–2.4 for samples S1200 and R1200 too, and the curves follow the same order (from up to down) in Figure 2a at each evaluation.

### 3.7 Regular Evaluations of the Experiments from 2018

**Figure 4:**
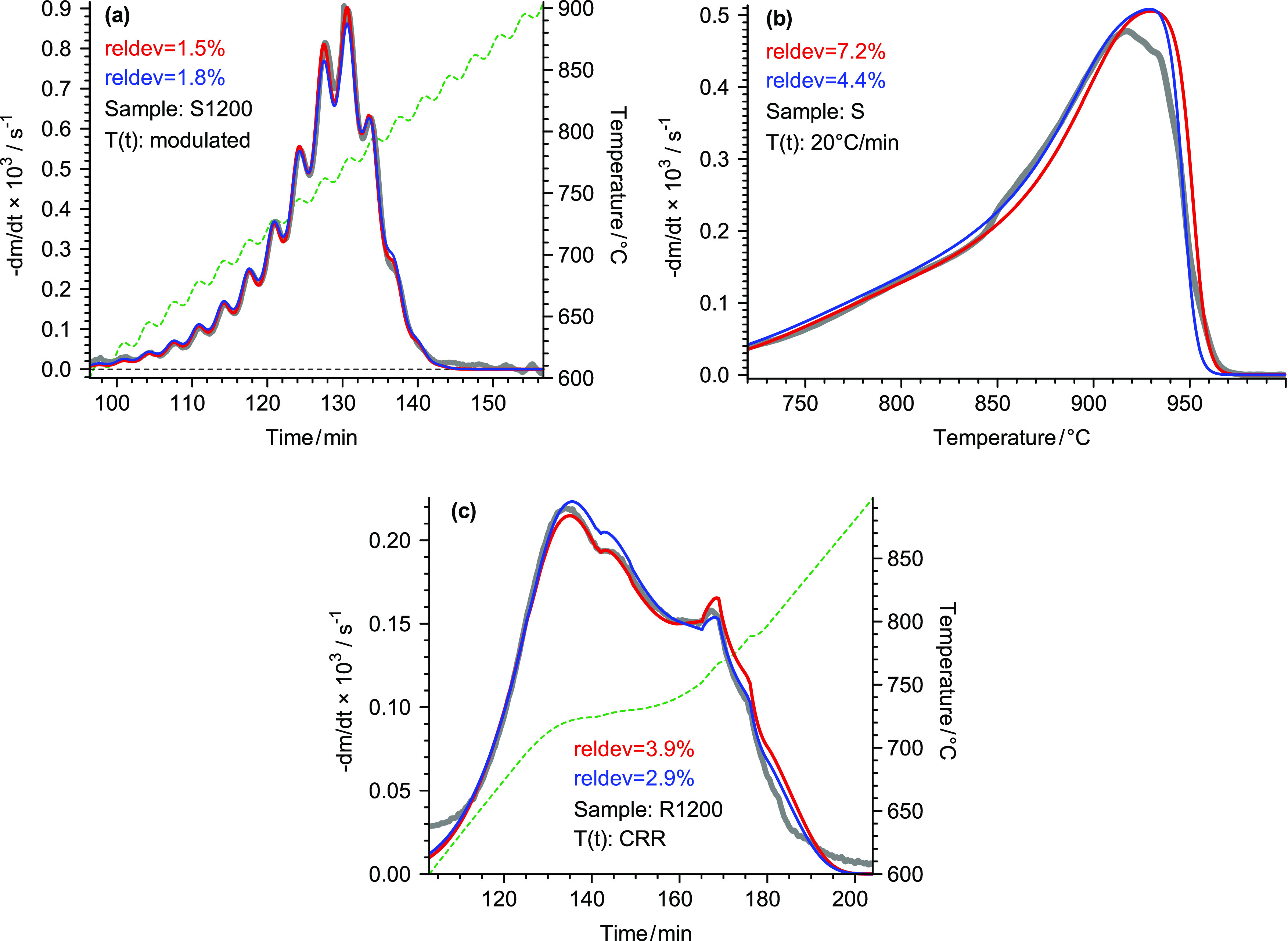
The best (a), the worst (b), and a typical fit quality (c) obtained by Evaluation 2.5. The corresponding (−d*m*/d*t*)^calc^ curves are denoted by red color. The (−d*m*/d*t*)^calc^ data of Evaluation 2.2 are indicated by blue lines for comparison. The thick gray lines and the green dashed lines represent the experimental curves and the observed *T*(*t*) data, respectively.

In Evaluation 2.5, all kinetic parameters were required to be identical for the experiments on a given sample. The fit quality worsened in this way: reldev_12_ increased from 3.0 to 4.2%. However, the latter is still a much better value than the reldev_12_ values presented in Sections 3.1–3.4 on other charcoals. Figure 4 displays the best, the worst, and a typical fit quality obtained by this evaluation. For comparison, the (−d*m*/d*t*)^calc^ curves from Evaluation 2.2 are also shown by blue lines.

The next two rows in Table 2 contain test evaluations similar to the test evaluation described in the previous section. Accordingly, the determination of the model parameters was based on the linear and modulated *T*(*t*) experiments in Evaluation 2.6 and on the modulated and CRR experiments in Evaluation 2.7. These evaluations gave practically the same *E* as Evaluation 2.5. The obtained *f*(*X*) functions were nearly identical in Evaluations 2.5–2.7, as shown in Figure 3b.

Comparing Figure 3b to 3a, one can notice the identical order of the curves (from up to down), a similarity in the shapes of the curves, and the closeness of the curves of Samples S and R in both Figure 3a,b.

## 4 Discussion

### 4.1 Note on the Activation Energy Values

It is difficult to compare the resulting *E* values to the values published in the literature because a very wide range of activation energies have been reported. In the recent literature, one can find *E* values from 15 up to 544 kJ/mol.^28,29^ Note that the activation energy is not supposed to be a formal parameter that may have any values. According to the IUPAC definition, the activation energy is “an empirical parameter characterizing the exponential temperature dependence of the rate coefficient”.^30^

### 4.2 About the Modeling Approaches Used in This Work

The first two lines of Tables 1 and 2 indicate that the present model resulted in similar activation energies as the earlier evaluations of the same experiments with other models,^5,6^ while the fit quality improved. Technically, the present evaluations were not more complicated than the ones in the work of Wang et al., 2013,^5^ and they were much simpler than the evaluations with two partial peaks in the study of Wang et al., 2018.^6^ The advantage of the presented approaches lies in the versatility of the polynomial approximations. As mentioned earlier, the real chars and charcoals may be composed of parts with different reactivities, and the presence of the mineral matter may also complicate their gasification from a kinetic point of view.

Part of the evaluations was carried out so that a kinetic parameter was allowed to scatter from experiment to experiment. As outlined in Section 3.3, it is a way to describe formally the scatter in the sampling from an inhomogeneous substance for the experiments; the experimental errors of the experiments in the thermal analysis; and the different thermal deactivation of the chars during the different *T*(*t*) programs.^6,16,31^

A typical nonisothermal study in the field is based only on linear *T*(*t*) experiments. In such cases, the evaluation with varying *A* may be an ill-defined problem because the *m*(*t*) and −d*m*/d*t* curves are similar to each other at linear *T*(*t*) programs with different heating rates. The extent of their distance is characteristic to *E* and has been used for the determination of *E* for decades.^32,33^ A scatter of *A* can counterbalance or can even completely eliminate the dependence of the linear *T*(*t*) experiments on the heating rate. However, the studies of the present authors included nonlinear *T*(*t*) programs as well.^5,6,16,31^ Three experiments were available for each sample at each CO_2_ concentration that were carried out at a linear, a CRR and a modulated *T*(*t*), as outlined in Sections 2.1, 3.1, and 3.5. The heating rate was (obviously) constant at the linear *T*(*t*), while it varied during the CRR experiments. The modulated experiments contained heating and cooling sections in the experiments of Wang et al., 2018,^6^ as the green dashed line in Figure 4a shows, while periods of nearly zero and higher heating rates occurred in the modulated experiments of Wang et al., 2013,^5^ as shown in Figure 1a,c. The use of the nonlinear *T*(*t*) programs made possible the evaluations with varying *A*. The differences between the results of the evaluations with varying *A* and nonvarying *A* were moderate as the tables and figures show. The activation energy showed a change of 9% as discussed in Section 3.3 and 15% as discussed in Sections 3.6 and 3.7. At this moment, we cannot decide which of the two employed approaches is more exact. The test evaluations with fewer experiments indicated that the employed experiments contained sufficient information for both types of approximations.

### 4.3 About the *f*(*X*) Functions

As mentioned above, the various approaches used in this work resulted in *f*(*X*) functions with similar shapes and identical arrangement, as seen in Figures 2 and 3. A closer look on the model parameters revealed that the fourth-order and fifth-order terms in the Chebyshev series were small. (See the data presented in the Supporting Information.) The ratio of the fourth-order and fifth-order terms to the maximum of the corresponding *p*(*x*) varied between 0.001 and 0.007. This indicates that the use of fifth-order polynomials did not lead to ill-conditioning in the calculations: the superfluous fourth- and fifth-order polynomial terms decreased to low values instead of superposing unnecessary flutters onto the calculated curves. The results indicate that the experiments reevaluated in the present work could have been approximated by third-order *p*(*x*) polynomials instead of fifth-order polynomials. Earlier, we had a different experience with this type of modeling on biomass pyrolysis and biomass combustion where the use of fifth-order or even higher-order polynomials was found to be optimal.^18,19^ Note that the expansion by Chebyshev polynomials is an optimal way for the truncation of polynomials in a minimax sense; hence, the real importance of the fifth- and fourth-order terms can be judged better from eq 14 than from eq 12.^22,34^

## 5 Conclusions

A two-year-old kinetic model^18^ was found to be well suited for the kinetic description of char gasification experiments. Particular care was taken to check that the information content of the evaluated experiments is sufficient for the reliable determination of the model parameters. The main points of the work were as follows:

(1) The performance of the model was tested by the reevaluation of 24 TGA experiments from earlier publications. The adjustable parameters of the model were determined by the method of least squares by evaluating groups of experiments together. The procedure aimed at finding the best-fitting models for the normalized mass loss rate, (−d*m*/d*t*)^obs^. The change of the reactivity during the progress of the reactions was described by using a suitable approximation for the *Af*(*X*) term of eq 1.
(2) Two evaluation strategies were tested. In the regular evaluations, the same kinetic parameters were employed for the experiments of a given sample. The use of experiments with modulated and CRR temperature programs (more precisely, the higher information content of the series of experiments containing such temperature programs) made it possible to employ another approach too, when the preexponential factor was allowed to vary from experiment to experiment. The latter approach allows a formal description of the differences of the experiments caused by the different thermal deactivation in the different experiments and by some inevitable systematic errors of the TGA experiments. Contrary to our earlier works, we carried out both approaches for the evaluations of the present study and compared the results. Moderate differences were found in the *E* values and in the *f*(*X*) functions. Further studies are needed to determine which of the two approaches provides the more accurate results.
(3) The present evaluations resulted in practically the same activation energies as the ones in the original publications, as the first two rows show in both Tables 1 and 2, while the fit quality increased. Technically, the present evaluations were not more difficult than our earlier evaluations by the method of least squares, and they were much simpler than the evaluations with two partial peaks in the study of Wang et al., 2018.^6^ Accordingly, the use of eq 3 as an empirical model can be advised for kinetic studies dealing with the CO_2_ gasification of chars.
(4) The evaluations were based on 12 experiments. As a test, each evaluation of the study was repeated with only 8 experiments. The results of these calculations indicated that the information content of the employed experiments is sufficient for the evaluation approaches of this work.

## Supporting Information Available

The obtained kinetic parameters for each evaluation based on 12 experiments and explanations for the calculation methods of the paper (PDF)

## Nomenclature

*A*: preexponential factor [s^–1^]
*a*, *b*: coefficients in the polynomial approximations of *Af*(*X*) [ln s^–1^]
*c*: coefficient in eq 7 [dimensionless]
*C*_CO_2__: = volume concentration of CO_2_ [dimensionless]
*E*: activation energy [kJ/mol]
*f*(*X*): function in eq 1 [dimensionless]
*h*_*j*_: height of an experimental −d*m*/d*t* curve in eq 6 [s^–1^]
*m*: the mass of the sample normalized by the initial dry sample mass [dimensionless]
*of*: objective function minimized by the method of least squares [dimensionless]
*N*_exper_: number of the experiments evaluated together by the method of least squares
*N*_*j*_: number of the evaluated data on the *j*th experimental curve
ν: reaction order with respect to *P*_CO_2__ [dimensionless]
*p*(*X*), *p*(*x*): fifth-order polynomials [ln s^–1^]
*R*: gas constant (8.3143 × 10^–3^ kJ mol^–1^ K^–1^)
reldev: the deviation between the observed and calculated data expressed as the percent of the corresponding peak height [%]
reldev_*N*_: rms of the reldev values of *N* experiments [%]
*t*: time [s]
*T*: temperature [°C, K]
*T*_1_(*x*)...*T*_5_(*x*): Chebyshev polynomials of the first kind
*X*: conversion (reacted fraction)
*x*: 2*X* – 1 [dimensionless]

## Subscripts

*i*: digitized point on an experimental curve
*j*: experiment
