# Supplementary material for: Empirical Kinetic Models for the CO2 Gasification of Biomass Chars. Part 1. Gasification of Wood Chars and Forest Residue Chars
Source: ACS Omega. 2021 Oct 10;6(41):27552–60. doi: 10.1021/acsomega.1c04577 (PMC8529891; doi:10.1021/acsomega.1c04577)
Supplement: Supplementary file 1 — ao1c04577_si_001.pdf [file ao1c04577_si_001.pdf]

Supporting Information for Article

Empirical Kinetic Models for the CO_2 Gasification of Biomass Chars. Part 1. Gasification of Wood Chars and Forest Residue Chars

Gábor Várhegyi[†,*] • Liang Wang[‡] • Øyvind Skreiberg[‡]

[†] Institute of Materials and Environmental Chemistry, Research Centre for Natural Sciences, PO Box 286, Budapest, Hungary 1519

[*] corresponding author, email: varhegyi.gabor@ttk.hu or gvarhegyi@gmail.com

[‡] SINTEF Energy Research, Postboks 4761 Torgarden, NO-7465 Trondheim, Norway

Keywords: Least Squares • Kinetics • Non-isothermal • Charcoal • Biomass • Wood.

Scope of this Document: Models are presented for the CO_2 gasification of 6 charcoals. The performance of the models was tested by the reevaluation of TGA experiments belonging to earlier publications. Besides, a brief explanation is given on the parameter transformations that facilitate the least squares evaluations.

Contents

1. Simple parameter transformations to facilitate the least squares evaluations S2
2. Gasification of wood and forest residue chars at two CO_2 concentrations S5
4. Gasification of chars from slow and fast pyrolysis S8

1. Simple parameter transformations to facilitate the least squares evaluations

1.1. The kinetic model. First the employed empirical model is summarized here for the convenience of the reader. The kinetic differential equation has the form

$$dX/dt = A f(X)\, e^{-E/(RT)} \qquad \text{(S1)}$$

where X is the reacted fraction (conversion) and $Af(X)$ is an empirical function. Obviously $Af(X)$ can be factorized to an A and an $f(X)$ in endless ways. In the present work we normalized $f(X)$ so that its height is 1 at X=0. The A values listed in this document were calculated by this assumption.

$Af(X)$ is approximated by using a polynomial as follows:

$$A f(X) = e^{p(X)} (1-X) \qquad \text{(S2)}$$

where the term (1-X) ensures that dX/dt would be 0 when X=1 for any $p(X)$ polynomial.

In this way eq S1 has the form

$$dX/dt = e^{p(X)-E/(RT)} (1-X) \qquad \text{(S3)}$$

1.2 Simple parameter transformations. Fifth order polynomials were used in the present work. Their coefficients were determined by optimizing the fit between the experimental and measured data. X varies between 0 and 1. In this interval the higher powers of X are rather similar to each other, and their coefficients can highly compensate each other. This cause severe technical problems during the determination of the corresponding polynomial coefficients. The problem can easily be mitigated by transforming the X values into the [–1,1] interval:

$$x = 2X - 1 \qquad \text{(S4)}$$

The situation is illustrated by Figure S1 that shows the powers of x. The higher powers are similar to each other in the [0,1] interval. For example, x^4 can be approximated well by a linear combination of x^3 and x^5 when $0 \le x \le 1$. (See the orange-colored dots in Figure S1.) This is not so when $x<0$ because x^3 and x^5 are negative while x^4 is positive at $x<0$. With this transformation p has the form

$$p(x) = a_0 + a_1 x + a_2 x^2 + a_3 x^3 + a_4 x^4 + a_5 x^5 \qquad \text{(S5)}$$

The a_0 … a_5 coefficients can be determined by the method of least squares. The interdependence of the polynomial coefficients can be further decreased if $p(x)$ is expressed by Chebyshev polynomials of the first kind, because these polynomials differ very much from each other. (See Figure S2 below). Accordingly, $p(x)$ can be rewritten as

$$p(x) = b_0 + b_1 T_1(x) + b_2 T_2(x) + b_3 T_3(x) + b_4 T_4(x) + b_5 T_5(x) \qquad \text{(S6)}$$

Using eq S6, a change of coefficient b_4, for example, are never compensated by the change of the other b coefficients during the numerical minimization of the least squares sum.

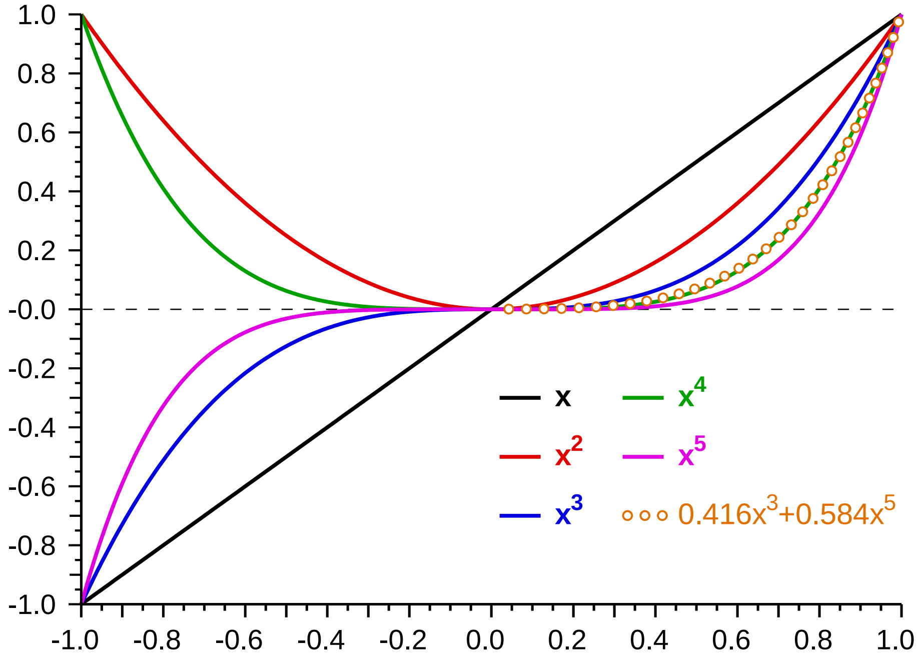

Figure S1. The powers of x in interval [-1,1]. The orange-colored dots illustrate a "compensation effect" (interdependence): a linear combination of x^3 and x^5 can approximate well x^4 when $0 \le x \le 1$. This interdependence was mitigated by transforming replacing X by x in the evaluations.

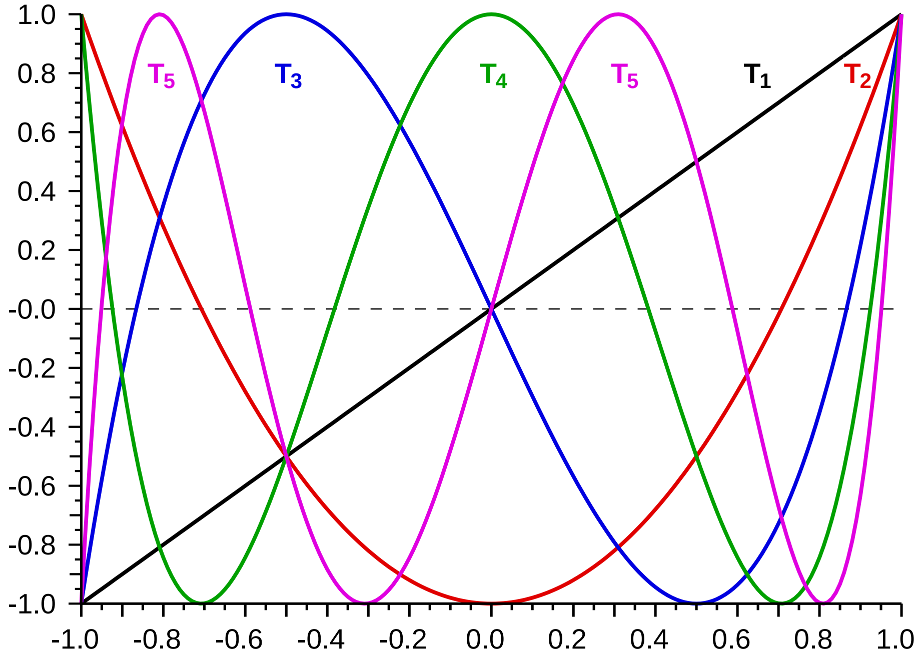

Figure S2. Chebyshev polynomials of the first kind in interval $-1 \le x \le 1$.

1.3. There is no need to transform back the obtained *p(x)* polynomials into *p(X)*. A *p(x)* expressed by eq S6 can as easily be used for any practical purpose. The values of the Chebyshev polynomials for eq S6 can easily be calculated by the well-known recurrence formula:

$$T_0 = 1 \tag{S7}$$

$$T_1 = x \tag{S8}$$

$$T_2 = 2xT_1 - T_0 \tag{S9}$$

…

$$T_n = 2xT_{n-1} - T_{n-2} \tag{S10}$$

Nevertheless, we presented the results both in the form of eq S5 and eq S6 in the present document. The formulas to convert a *p(x)* from eq S6 to eq S5 can be found in the literature or can be deduced easily. However, we did not transform back the *p(x)* polynomials to a series of the powers of *X* because a *p(X)* polynomial does not have any advantage over a *p(x)* polynomial.

2. Gasification of wood and forest residue chars at two CO_2 concentrations

The experiments belong to this work:

Wang, L.; Sandquist, J.; Várhegyi, G.; Matas Güell, B.: CO_2 Gasification of Chars Prepared from Wood and Forest Residue. A Kinetic Study. *Energy Fuels* **2013**, *27,* 6098-6107. *doi:* 10.1021/ef401118f Repository

The experiments were carried out at C_{CO_2}=0.6 and C_{CO_2}=1, where C_{CO_2} is the volume concentration of CO_2. Three heating programs were employed: 10°C/min, modulated *T(t)* and constant reaction rate *T(t)* (CRR). Figure 1 of the present article illustrates the fit quality belonging to the model data presented below. The corresponding *f(X)* functions are shown by solid lines in Figure 2 of the article.

Evaluation 1.2 (regular evaluation based on 12 experiments)

E=220

(i) Wood char

$p(x)= \mathbf{a_0} +0.6640x +0.1397x^2 -0.4879x^3 +0.3938x^4 +0.9513x^5$

Expressed by Chebyshev polynomials of the first kind:

$p(x)= \mathbf{b_0} +0.8927T_1(x) +0.2667T_2(x) +0.1753T_3(x) +0.0492T_4(x) +0.0595T_5(x)$

C_{CO_2}=0.6:

a_0=16.7033	b_0=16.9208	
$log_{10}A$= 6.9962	ln A=16.1093	A= 9.9128E+06

C_{CO_2}=1:

a_0=17.2280	b_0=17.4455	
$log_{10}A$= 7.2241	ln A=16.6340	A= 1.6752E+07

(ii) Forest residue char

$p(x)= \mathbf{a_0} +0.5021x +0.3342x^2 -0.0775x^3 +0.1628x^4 +0.5100x^5$

Expressed by Chebyshev polynomials of the first kind:

$p(x)= \mathbf{b_0} +0.7628T_1(x) +0.2485T_2(x) +0.1400T_3(x) +0.0204T_4(x) +0.0319T_5(x)$

C_{CO_2}=0.6:

a_0=17.1295	b_0=17.3577	
$log_{10}A$= 7.2492	ln A=16.6919	A= 1.7750E+07

C_{CO_2}=1:

a_0=17.3880	b_0=17.6161	
$log_{10}A$= 7.3614	ln A=16.9504	A= 2.2985E+07

Evaluation 1.4 (evaluation with varying *A* based on 12 experiments)

E=200

(i) Wood char

p(x)= **a_0** +0.7391x +0.1234x^2 -0.5833x^3 +0.4263x^4 +1.0931x^5

Expressed by Chebyshev polynomials of the first kind:

p(x)= **b_0** +0.9849T_1(x) +0.2748T_2(x) +0.1958T_3(x) +0.0533T_4(x) +0.0683T_5(x)

C_{CO_2}=0.6, 10°C/min

a_0=14.6358 b_0=14.8573

$\log_{10}$A= 6.0525 ln A=13.9365 A= 1.1286E+06

C_{CO_2}=0.6, modulated *T(t)*

a_0=14.6579 b_0=14.8795

$\log_{10}$A= 6.0622 ln A=13.9586 A= 1.1539E+06

C_{CO_2}=0.6, CRR *T(t)*

a_0=14.4060 b_0=14.6276

$\log_{10}$A= 5.9528 ln A=13.7067 A= 8.9693E+05

C_{CO_2}=1, 10°C/min

a_0=15.0681 b_0=15.2896

$\log_{10}$A= 6.2403 ln A=14.3688 A= 1.7389E+06

C_{CO_2}=1, modulated *T(t)*

a_0=15.0959 b_0=15.3175

$\log_{10}$A= 6.2524 ln A=14.3966 A= 1.7880E+06

C_{CO_2}=1, CRR *T(t)*

a_0=14.8833 b_0=15.1048

$\log_{10}$A= 6.1600 ln A=14.1840 A= 1.4455E+06

(ii) Forest residue char

p(x)= $\mathbf{a_0}$ +0.6019x +0.3360x^2 -0.1973x^3 +0.2288x^4 +0.7313x^5

Expressed by Chebyshev polynomials of the first kind:

p(x)= $\mathbf{b_0}$ +0.9110T_1(x) +0.2824T_2(x) +0.1792T_3(x) +0.0286T_4(x) +0.0457T_5(x)

C_{CO_2}=0.6, 10°C/min

a_0=15.0356 b$_0$=15.2894

$\log_{10}$A= 6.2818 ln A=14.4644 A= 1.9135E+06

C_{CO_2}=0.6, modulated *T(t)*

a_0=14.9210 b_0=15.1748

$\log_{10}$A= 6.2321 ln A=14.3499 A= 1.7064E+06

C_{CO_2}=0.6, CRR *T(t)*

a_0=14.8127 b_0=15.0665

$\log_{10}$A= 6.1850 ln A=14.2416 A= 1.5312E+06

C_{CO_2}=1, 10°C/min

a_0=15.4130 b_0=15.6668

$\log_{10}$A= 6.4458 ln A=14.8419 A= 2.7910E+06

C_{CO_2}=1, modulated *T(t)*

a_0=15.3793 b_0=15.6331

$\log_{10}$A= 6.4311 ln A=14.8081 A= 2.6983E+06

C_{CO_2}=1, CRR *T(t)*

a_0=14.9348 b_0=15.1886

$\log_{10}$A= 6.2380 ln A=14.3636 A= 1.7300E+06

4. Gasification of chars from slow and fast pyrolysis

The experiments belong to this work:

Wang, L.; Li, T.; Várhegyi, G.; Skreiberg, Ø.; Løvås, T. CO_2 Gasification of chars prepared by fast and slow pyrolysis from wood and forest residue. A kinetic study. *Energy Fuels* **2018**, *32*, 588-597 *doi:* 10.1021/acs.energyfuels.7b03333 Repository

In this section S and R denotes chars that formed from spruce and forest residue, respectively, in the TGA apparatus during the gasification experiments. S1200 and R1200 denotes spruce and forest residue chars prepared by fast pyrolysis. Three heating programs were employed: 10°C/min, modulated *T(t)* and constant reaction rate *T(t)* (CRR). Figure 4 in the present article illustrates the fit quality belonging to the data presented below. The corresponding *f(X)* functions are shown by solid lines in Figure 3 in the article.

Evaluation 2.5 (regular evaluation based on 12 experiments)

E=266

(i) Sample S

$p(x)= 22.0167 +0.3275x +0.7014x^2 -1.1871x^3 +1.1091x^4 +0.7033x^5$

Expressed by Chebyshev polynomials of the first kind:

$p(x)= 22.7833 -0.1232T_1(x) +0.9052T_2(x) -0.0770T_3(x) +0.1386T_4(x) +0.0440T_5(x)$

$\log_{10}A=10.4159$ ln A=23.9834   A= 2.6053E+10

(ii) Sample R

$p(x)= 22.5157 +0.1343x +1.1515x^2 -0.8785x^3 +0.3517x^4 +0.2034x^5$

Expressed by Chebyshev polynomials of the first kind:

$p(x)= 23.2234 -0.3974T_1(x) +0.7516T_2(x) -0.1561T_3(x) +0.0440T_4(x) +0.0127T_5(x)$

$\log_{10}A=10.6662$ ln A=24.5597   A= 4.6362E+10

(iii) Sample S1200

$p(x)= 24.7924 +0.3931x -0.2766x^2 -0.1669x^3 +0.4004x^4 -0.4314x^5$

Expressed by Chebyshev polynomials of the first kind:

$p(x)= 24.8043 -0.0016T_1(x) +0.0619T_2(x) -0.1765T_3(x) +0.0501T_4(x) -0.0270T_5(x)$

$\log_{10}A=10.9101$ ln A=25.1214   A= 8.1298E+10

(iv) Sample R1200

$p(x)= 24.4588 -0.0591x -0.1670x^2 -0.2720x^3 +0.2163x^4 -0.7263x^5$

Expressed by Chebyshev polynomials of the first kind:

$p(x)= 24.4564 -0.7170T_1(x) +0.0247T_2(x) -0.2949T_3(x) +0.0270T_4(x) -0.0454T_5(x)$

$\log_{10}A=11.1029$ ln A=25.5654   A= 1.2675E+11

Evaluation 2.2 (evaluation with varying *A* based on 12 experiments)

E=231

(i) Sample S

$p(x)= \mathbf{a_0} +0.4729x +0.6027x^2 -0.9847x^3 +1.0490x^4 +0.7666x^5$

Expressed by Chebyshev polynomials of the first kind:

$p(x)= \mathbf{b_0} +0.2135T_1(x) +0.8259T_2(x) -0.0066T_3(x) +0.1311T_4(x) +0.0479T_5(x)$

Sample S, 20°C/min

a_0=18.5030 b_0=19.1978

$\log_{10}A$= 8.6424 ln A=19.9000 A= 4.3898E+08

Sample S, modulated *T(t)*

a_0=18.2435 b_0=18.9382

$\log_{10}A$= 8.5297 ln A=19.6404 A= 3.3863E+08

Sample S, CRR *T(t)*

a_0=18.0801 b_0=18.7749

$\log_{10}A$= 8.4588 ln A=19.4771 A= 2.8759E+08

(ii) Sample R

$p(x)= \mathbf{a_0} +0.3127x +1.0361x^2 -0.8508x^3 +0.2763x^4 +0.4006x^5$

Expressed by Chebyshev polynomials of the first kind:

$p(x)= \mathbf{b_0} -0.0750T_1(x) +0.6562T_2(x) -0.0875T_3(x) +0.0345T_4(x) +0.0250T_5(x)$

Sample R, 20°C/min

a_0=18.8245 b_0=19.4462

$\log_{10}A$= 8.8051 ln A=20.2744 A= 6.3838E+08

Sample R, modulated *T(t)*

a_0=18.6629 b_0=19.2845

$\log_{10}A$= 8.7349 ln A=20.1128 A= 5.4310E+08

Sample R, CRR *T(t)*

a_0=18.5699 b_0=19.1915

$\log_{10}A$= 8.6945 ln A=20.0198 A= 4.9487E+08

(iii) Sample S1200

$p(x)= \mathbf{a_0} +0.5343x -0.2155x^2 -0.2985x^3 +0.2757x^4 -0.1036x^5$

Expressed by Chebyshev polynomials of the first kind:

$p(x)= \mathbf{b_0} +0.2457T_1(x) +0.0301T_2(x) -0.1070T_3(x) +0.0345T_4(x) -0.0065T_5(x)$

Sample S1200, 20°C/min

a_0=20.8152 b_0=20.8109

$\log_{10}A$= 9.0087 ln A=20.7433 A= 1.0202E+09

Sample S1200, modulated *T(t)*

a_0=20.6765 b_0=20.6722

$\log_{10}A$= 8.9485 ln A=20.6046 A= 8.8808E+08

Sample S1200, CRR *T(t)*

a_0=20.5741 b_0=20.5698

$\log_{10}A$= 8.9040 ln A=20.5022 A= 8.0164E+08

(iv) Sample R1200

$p(x)= \mathbf{a_0} +0.0760x -0.1563x^2 -0.1332x^3 +0.1490x^4 -0.5654x^5$

Expressed by Chebyshev polynomials of the first kind:

$p(x)= \mathbf{b_0} -0.3773T_1(x) -0.0036T_2(x) -0.2100T_3(x) +0.0186T_4(x) -0.0353T_5(x)$

Sample R1200, 20°C/min

a_0=20.4556 b_0=20.4333

$\log_{10}A$= 9.1510 ln A=21.0709 A= 1.4158E+09

Sample R1200, modulated *T(t)*

a_0=20.3714 b_0=20.3491

$\log_{10}A$= 9.1144 ln A=20.9867 A= 1.3014E+09

Sample R1200, CRR *T(t)*

a_0=20.3339 b_0=20.3116

$\log_{10}A$= 9.0981 ln A=20.9493 A= 1.2536E+09
